# Molecular Docking and Absorption, Distribution, Metabolism, and Excretion (ADME) Analysis: Examining the Binding Modes and Affinities of Myricetin With Insulin Receptor, Glycogen Synthase Kinase, and Glucokinase

**DOI:** 10.7759/cureus.53810

**Published:** 2024-02-07

**Authors:** Ramadurai Murugan, Silambarasan Tamil Selvan, Mukesh Kumar Dharmalingam Jothinathan, Guru Prasad Srinivasan, Remya Rajan Renuka, Monisha Prasad

**Affiliations:** 1 Center for Global Health Research, Saveetha Medical College and Hospital, Saveetha Institute of Medical and Technical Sciences, Chennai, IND

**Keywords:** lipinski, therapeutics, auto dock, molecular docking, myricetin, diabetes

## Abstract

Aim

By using molecular docking analysis (MDA) to examine its interactions with important regulatory proteins linked to diabetes, such as glycogen synthase kinase 3 beta (GSK3β), insulin receptor (IR), and glucose kinase (GCK), this study seeks to explore the therapeutic potential of myricetin, a naturally occurring flavonoid.

Objective

The main goal is to determine potential effects on insulin signalling, GSK3β activity, and glucose metabolism by evaluating the binding affinities of myricetin with GCK, IR, and GSK3β through MDA. In order to assess the drug affinity of myricetin, the study also intends to perform absorption, distribution, metabolism, and excretion (ADME) studies.

Materials and methods

To model the interaction between myricetin and the target proteins (GCK, IR, and GSK3β), we used molecular docking analysis with computational tools. ADME studies were also included in the study to evaluate drug affinity. Identification of binding sites, essential residues, and interaction stability were all part of the structural analysis.

Results

As evidence of possible interactions with these regulatory proteins, myricetin showed positive binding affinities with GCK, IR, and GSK3β. Strong interactions with important ligand recognition residues were seen in the docking into IR, indicating a potential impact on insulin signalling. Moreover, a strong binding affinity for GCK indicated potential effects on the metabolism of glucose. Studies using ADME confirmed the high drug affinity of myricetin.

Conclusion

This work sheds light on the multi-target potential of myricetin in the regulation of diabetes. It appears that it has the ability to influence glucose metabolism, suppress GSK3β activity, and regulate insulin signalling based on its interactions with IR, GSK3β, and GCK. Although these computational results show promise, more experimental work is necessary to confirm and fully understand the precise mechanisms that underlie myricetin's effects on the regulation of diabetes.

## Introduction

Diabetes mellitus, a prevalent non-communicable disease, shares similarities with hypertension and affects a substantial proportion of the global population. This endocrine disorder results from either insufficient pancreatic production of insulin or the body's inefficient use of insulin [1]. Diabetes mellitus affects around 6% of the global population, with a higher incidence among older individuals. Diabetes is divided into two categories: Type 1 and Type 2. When the body is unable to produce enough insulin, type 1 diabetes develops, requiring lifelong insulin administration. On the other hand, Type 2 diabetes results from insulin resistance in the body's cells and is frequently misdiagnosed for an extended length of time. Increasing awareness of diabetes mellitus is essential because managing the condition early on can prevent complications from arising [2]. Timely screenings, accurate diagnosis, and appropriate treatment strategies are essential for people with diabetes to maintain healthy blood glucose levels and overall well-being. By promoting understanding, emphasizing regular check-ups, and encouraging adherence to treatment plans, we can effectively support individuals in managing their diabetes and reducing the risk of complications [3]. The significance of public education and healthcare initiatives cannot be overstated in addressing the growing burden of diabetes mellitus. By raising awareness, promoting preventive measures, and advocating for early intervention, we can mitigate the impact of diabetes on individuals, families, and communities. A comprehensive healthcare approach, including regular screenings and access to quality treatment, is crucial in reducing the prevalence and impact of diabetes mellitus worldwide [4].

The natural flavonoid myricetin, which can be found in many plant-based foods, has demonstrated promise in helping to manage diabetes. Antihyperglycemic properties of myricetin have been discovered. By increasing insulin sensitivity and encouraging glucose uptake in cells, it can aid in blood glucose regulation. Myricetin may act by activating signalling pathways associated with glucose metabolism, such as the adenosine monophosphate-activated protein kinase (AMPK) pathway, which is crucial for maintaining glucose homeostasis [5]. It has been demonstrated that myricetin stimulates pancreatic beta cells to secrete insulin. People with diabetes, particularly those with reduced insulin production, may benefit from this. Myricetin may aid in glycaemic control and general glucose regulation by increasing insulin secretion [6]. Strong anti-inflammatory and antioxidant characteristics of myricetin may help lessen the negative effects of inflammation and oxidative stress in diabetics. Myricetin may enhance insulin sensitivity, shield pancreatic beta cells from damage, and avert diabetes-related complications by lowering oxidative stress and inflammation [7].

Molecular docking analysis (MDA) is a widely utilized computational technique for structure-based drug design [8]. It plays a pivotal role in unravelling the interactions between small molecules, such as myricetin, and larger macromolecules involved in diseases like diabetes. In our study, our focus was to investigate the molecular interactions between myricetin and key regulators implicated in diabetes, including glycogen synthase kinase 3 beta (GSK3β), insulin receptor (IR), and glucose kinase (GCK). Through the application of molecular docking, our aim was to elucidate the binding modes and potential therapeutic effects of myricetin with these regulators at a molecular level. The analysis provides valuable insights that could inform future efforts in the field of drug design and optimisation related to diabetes management. It also advances our understanding of the potential therapeutic effects of myricetin [9-12].

## Materials and methods

Protein preparation

We used the Protein Data Bank (PDB) and their corresponding Protein Data Bank identity numbers, R (1IR3): Insulin Receptor, GSK3β (3F7Z): Glycogen synthase kinase-3 beta, and GCK (4IXC): Glucokinase. With the aid of a Python molecule viewer tool (Python Software Foundation, Wilmington, DE, USA), these protein structures were ready for the ensuing MDA analyses. Our ability to eliminate ligands and water ions with this software meant that only the protein component was left for additional examination. The docking process was made much simpler by concentrating only on the interactions between the drug and the protein (or ligand), thanks to this crucial step. IR, GSK3β, and GCK substrate-binding domains were examined using Discovery Studio 4.5 (BIOVIA, San Diego, CA, USA) to determine the active site residues. The ability to identify the precise protein residues that are essential for ligand binding and interaction was made possible by this software. Determining these residues in the active site was crucial to comprehending putative binding sites and forecasting how ligands and protein targets would interact.

Ligand preparation

Our main goal in this work was to examine the molecular interactions of myricetin (Compound ID: 5281672) using molecular docking analysis. We started the investigation by obtaining the three-dimensional (3D) structure of myricetin from PubChem, a comprehensive chemical database that is frequently used by scientists. We used the SWISS-ADME prediction tools to evaluate the drug-like qualities of myricetin. These instruments offered insightful information about significant drug-related characteristics like drug-likeness, lipophilicity, and solubility. This assessment played a crucial role in evaluating the potential of myricetin as a therapeutic compound.

Molecular docking analysis

To prepare the 3D structures of the compounds, including myricetin, for the subsequent MDA simulations, we utilized AutoDock Tools (ADT) software version 1.56 (Center for Computational Structural Biology, La Jolla, CA, USA). ADT facilitated the processing and refinement of the structures, generating pdbqt files that are necessary for molecular interaction analysis. To create the essential grid maps, we established a box size of 90 × 90 × 90 xyz points, centered on the active site residues of IR, GSK3β, and GCK, utilizing AutoGrid, a component within AutoDock software. Our docking analysis employed the Lamarckian genetic algorithm, a method integrating Lamarckism and enabling modifications in genetic material across generations. By following these steps, we ensured the proper preparation of the 3D structures and conducted molecular docking to explore the molecular interactions between myricetin and the target proteins. This approach allowed us to gain valuable insights into the potential binding modes and interactions, contributing to our understanding of the therapeutic effects of myricetin in the context of our study.

ADME studies

Our study examined the ADME characteristics of myricetin using in silico pharmacokinetic analysis. As per Lipinski's 2001 guidelines [13], this required predicting several important parameters, including molecular weight, topological polar surface area (TPSA), miLog P, number of rotatable bonds, and the number of hydrogen donor and acceptor atoms. We used a web application (www.swissadme.ch) for this evaluation that can predict pharmacokinetic characteristics and compute physicochemical descriptors for small molecules [14]. We were able to learn about important drug properties on this platform, such as how drugs interact with particular biological barriers like P-glycoproteins, cytochromes P450, and the blood-brain barrier. Compounds that showed positive values in our investigation were recognised as being able to cross the blood-brain barrier (BBB) easily, which could affect the compound's distribution.

## Results

Molecular docking (MD)

Our goal was to use MD analysis to look at the interactions between IR, GSK3β, and GCK, three important proteins implicated in diabetes, and their regulatory activity. We learned a great deal about the binding affinities between myricetin and the diabetic regulating targets through MDA simulations. Our analysis is summarised in Table 1, which demonstrates the strong binding affinities between myricetin and the target proteins. With binding energies of -6.9 kcal/mol for IR, -6.2 kcal/mol for GSK3β, and -8.2 kcal/mol for GCK, myricetin exhibited robust interactions with these proteins.

**Table 1:** Molecular docking (MD) analysis

S. no	Drug	Protein	Binding energy (kcal/mol)	No. of H bonds involved	Amino acid residues
1.	Myricetin (5281672)	Insulin Receptor (IR33)	-6.9	3	GLU1108, ASP1143, & HIS1057
2.		Glycogen Synthase Kinase 3 Beta (GSK3β) (3F7Z)	-6.2	3	SER361, ARG340, & LYS1024
3.		Glucose Kinase (GCK) (4IXC)	-8.2	4	ASP1038, ARG937, PRO948, & ILE951

**Figure 1:**
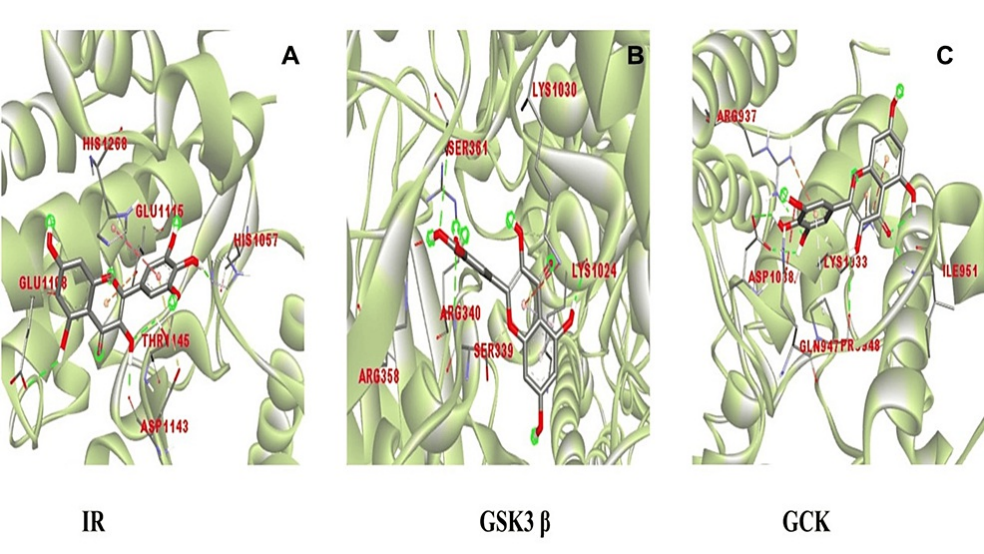
Molecular docking analysis of myricetin with diabetic regulating targets. A) Insulin receptor (IR), B) glycogen synthase kinase 3 beta (GSK3β), and C) glucose kinase (GCK).

The formation of hydrogen bonds between myricetin and particular active site residues within the target proteins, such as glutamic acid (GLU1108), aspartic acid (ASP1143), and histidine (HIS1057) for IR, serine (SER361), arginine (ARG340), and lysine (LYS1024) for GSK3β, and aspartic acid (ASP1038), arginine (ARG937), proline (PRO948), and isoleucine (ILE951) for GCK, as highlighted in Figure 1, which provided additional support for the docking analysis. These interactions were significant. Consequently, the compound's possible interactions with the active sites were brought to light. The stabilisation of myricetin's binding to the target proteins was greatly aided by these hydrogen bonds.

ADME properties

**Figure 2:**
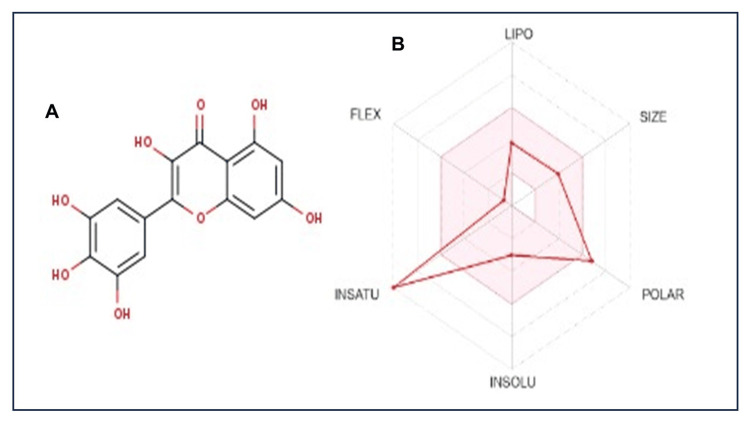
Physiological properties of myricetin. A) 2D structure of myricetin, B) Lipinski violation shown in red line.

Lipinski's rule of five serves as a guideline to assess the drug-likeness of chemical compound, myricetin particularly in determining their potential as orally active drugs in humans. In our investigation, myricetin displayed one violation concerning molecular mass, hydrogen donors, and acceptors among the five criteria outlined. We illustrated the Lipinski violations of myricetin in Figure 2. Predicting the transport characteristics of drug candidates through the BBB and within the intestines is largely dependent on TPSA. Myricetin, with a TPSA score of 151.59, displayed a notably high TPSA value, indicating its preference for a hydrophilic nature. The one violation of Lipinski's rule observed for myricetin imply a potential limitation in its oral bioavailability. Although no specific score was available, the high TPSA value of 151.59 suggests that myricetin's hydrophilic nature might hinder its easy passage through the BBB. The comparative ADME profile of myricetin can be found in Table 2.

**Table 2:** Absorption, Distribution, Metabolism, and Excretion (ADME) properties for myricetin

Physiochemical properties	
Structure	
Mol wt (g/mol)	318.24
Formula	C15H10O8
Canonical SMILES	Oc1cc(O)c2c(c1)oc(c(c2=O)O)c1cc(O)c(c(c1)O)O
Total Polar Surface Area (TPSA)	151.59
Blood-Brain Barrier (BBB) permeant	No
GI absorption	Low
Lipinski violations	1
Bioavailability Score	0.55
Synthetic Accessibility	3.27
Water solubility	0.314

## Discussion

Diabetes mellitus, a widespread non-communicable condition, bears resemblance to hypertension and impacts a significant segment of the global populace [3]. This endocrine disorder stems from inadequate insulin production by the pancreas or the body's inefficiency in utilizing insulin effectively. Adopting a comprehensive healthcare strategy that encompasses routine screenings and ensuring access to high-quality treatment is pivotal to mitigating the prevalence and repercussions of diabetes mellitus on a global scale [5].

Myricetin, a flavonoid belonging to the polyphenol group, has garnered significant interest in the scientific community due to its diverse and promising bioactive properties. Derived from various plant sources, such as fruits, vegetables, and medicinal herbs, myricetin has been recognized for its potent antioxidant capabilities The molecule's ability to neutralize reactive oxygen species and counteract oxidative stress positions it as a potential therapeutic agent in the context of various diseases, including neurodegenerative disorders and cardiovascular conditions [15,16]. Beyond its antioxidant properties, myricetin has exhibited anti-inflammatory effects, further contributing to its potential health benefits. Studies have suggested that myricetin may modulate inflammatory pathways, making it a subject of interest in the exploration of treatments for inflammatory conditions. Moreover, myricetin has demonstrated anti-cancer properties in preclinical investigations, showing promise in inhibiting the growth of cancer cells and impeding tumor progression [17]. While myricetin presents a spectrum of potential pharmacological effects, it is essential to consider its safety profile. Existing literature provides insights into the toxicity of myricetin, ensuring a holistic understanding of its pharmacological characteristics. Researchers and clinicians are increasingly exploring the therapeutic potential of myricetin, aiming to harness its bioactive properties for the development of novel treatments. As we delve into the specific interactions of myricetin with protein targets in our study, we aim to contribute to the growing body of knowledge surrounding this intriguing molecule and its potential applications in various health contexts.

Our investigation primarily focused on delving into the molecular interactions between myricetin, a commonly utilized compound in diabetes management, and the active sites of specific proteins. To accomplish this, we utilized the crystal structures of IR, GSK3β, and GCK as the fundamental framework for our analysis. Before initiating the MDA, we meticulously analyzed the binding sites within these proteins to ensure a comprehensive understanding. In this process, the protein conformation was fixed while the ligand's torsional degrees of freedom were relaxed. This method made it easier to evaluate possible binding sites and interactions between myricetin and the proteins that were the targets. By considering diverse orientations and relaxing the torsional degrees of freedom of myricetin while maintaining fixed protein conformation, our study aimed to yield valuable insights into the molecular interactions between myricetin and these proteins involved in diabetes regulation.

These findings significantly contribute to enhancing our understanding of the potential mechanisms underlying myricetin's therapeutic effects in diabetes treatment. Lipinski's rule of five serves as a fundamental guideline for assessing whether a chemical compound, such as myricetin, possesses properties conducive to being an orally energetic drug in individuals [18]. In our study, myricetin exhibited a single violation among the five criteria, particularly regarding molecular mass, hydrogen donors, and acceptors. The depiction of myricetin's Lipinski violations is illustrated in Figure 2, enabling a clear visual understanding of its compliance with these established criteria. TPSA plays a pivotal role in predicting a drug candidate's transport characteristics through biological barriers like the intestines and the BBB. Myricetin, with a TPSA score of 151.59, displayed a substantially high value, indicating its inclination towards a hydrophilic nature. The BBB is a highly selective and dynamic interface between the circulating blood and the central nervous system, comprising specialized endothelial cells, tight junctions, pericytes, and astrocytes. Its primary function is to regulate the passage of substances from the bloodstream into the brain, maintaining a tightly controlled microenvironment necessary for proper neuronal function [19]. In individuals with diabetes, there is increasing evidence to suggest that the BBB undergoes structural and functional alterations. Chronic hyperglycemia, oxidative stress, and inflammation associated with diabetes can compromise the integrity of the BBB, leading to increased permeability. This heightened permeability allows for the passage of inflammatory mediators, cytokines, and potentially harmful substances from the bloodstream into the brain, contributing to neuroinflammation and neuronal damage [20,21]. The impact of BBB dysfunction in diabetes extends beyond the realms of neuroinflammation. Disruptions in the BBB may also influence the transport of nutrients, hormones, and other signaling molecules crucial for brain function. Moreover, the compromised BBB may contribute to the development or exacerbation of cognitive impairments and neurodegenerative conditions often observed in diabetic patients, leading to neuropathy [22]. This characteristic suggests potential limitations for myricetin in crossing the BBB efficiently due to its hydrophilic properties compared to lipophilic compounds. The single violation observed in Lipinski's rule for myricetin may imply a potential constraint in its oral bioavailability, aligning with the criteria outlined by Lipinski. Although a specific BBB score was not available, the high TPSA value of 151.59 suggests that myricetin's hydrophilic nature might impede its straightforward passage across the BBB. For a comprehensive understanding of myricetin's pharmacokinetic profile, including its ADME characteristics, detailed information can be referenced in Table 2. This comparative analysis provides essential insights into myricetin's potential limitations as an orally active drug, particularly in terms of its bioavailability and BBB permeability, shedding light on considerations for its therapeutic application.

The observed significant binding affinities imply that myricetin may interact with the diabetic regulating targets to potentially inhibit inflammatory activity. This makes myricetin a potentially effective lead molecule to target the signalling pathways linked to diabetes. Myricetin interacts with IR, GSK3β, and GCK, which may enhance the effectiveness of diabetes treatments. These results support the investigation of myricetin as a potential therapeutic target for addressing the underlying causes of diabetes and promoting the creation of innovative therapies.

Limitation

The accuracy of algorithms and force fields may be a constraint on the study's reliance on molecular docking simulations. The results may not be as applicable in real life due to assumptions about proteins' static properties and oversimplifications in biological and pharmacokinetic models. A thorough knowledge of myricetin's interactions may be further limited by a lack of experimental validation and possible errors in ligand and receptor flexibility.

## Conclusions

Our study's main goal was to find out how the compound myricetin interacts with the important regulatory proteins, IR, GSK3β, and GCK, that are involved in diabetes. Finally, the binding attractions between myricetin and these diabetic targets using MDA. The strong drug biocompatibility of myricetin, which has anti-diabetic properties, is revealed by the ADME results. Our docking analysis yielded important results that showed myricetin had strong binding affinities with the diabetic regulatory proteins GSK3β, GCK, and IR. These findings suggest that myricetin may change the activity of these proteins through interactions, which may affect the processes associated with diabetes. All things considered, our study provides significant new insights into the molecular relationships between myricetin and diabetic regulatory proteins, emphasising the drug's potential effectiveness in modifying these targets' activities.

## References

[1] Aboul Enein BH, Bernstein J, Neary AC (2016). Dietary transition and obesity in selected arabic-speaking countries: a review of the current evidence. East Mediterr Health J.

[2] Rajput SA, Mirza MR, Choudhary MI (2020). Bergenin protects pancreatic beta cells against cytokine-induced apoptosis in INS-1E cells. PLoS One.

[3] Asif M (2014). The prevention and control the type-2 diabetes by changing lifestyle and dietary pattern. J Educ Health Promot.

[4] Venkatesh P (2022). Google lens: a potential cost-effective screening tool for diabetic retinopathy. Med Hypotheses.

[5] Borges LL, Martins FS, Franco JJ (2021). Effects of liquid extract from plinia cauliflora fruits residues on Chinese hamsters biochemical parameters. Braz J Biol.

[6] Dubey R, Kulkarni SH, Dantu SC (2021). Myricetin protects pancreatic β-cells from human islet amyloid polypeptide (hIAPP) induced cytotoxicity and restores islet function. Biol Chem.

[7] Tatipamula VB, Kukavica B (2021). Phenolic compounds as antidiabetic, anti-inflammatory, and anticancer agents and improvement of their bioavailability by liposomes. Cell Biochem Funct.

[8] Dos Santos RN, Ferreira LG, Andricopulo AD (2018). Practices in molecular docking and structure-based virtual screening. Methods Mol Biol.

[9] Lv K, Shao W, Pedroso MM (2021). Enhancing the catalytic activity of a GH5 processive endoglucanase from bacillus subtilis BS-5 by site-directed mutagenesis. Int J Biol Macromol.

[10] Naz R, Saqib F, Awadallah S (2023). Food polyphenols and type II diabetes mellitus: pharmacology and mechanisms. Molecules.

[11] Yang Y, Chen Z, Zhao X (2022). Mechanisms of kaempferol in the treatment of diabetes: a comprehensive and latest review. Front Endocrinol (Lausanne).

[12] Wu F, Shao Q, Xia Q (2021). A bioinformatics and transcriptomics based investigation reveals an inhibitory role of huanglian-renshen-decoction on hepatic glucose production of T2DM mice via PI3K/Akt/FoxO1 signaling pathway. Phytomedicine.

[13] Butina D, Segall M, Frankcombe K (2002). Predicting ADME properties in silico: methods and models. Drug Discov Today.

[14] Daina A, Michielin O, Zoete V (2017). SwissADME: a free web tool to evaluate pharmacokinetics, drug-likeness and medicinal chemistry friendliness of small molecules. Sci Rep.

[15] Semwal DK, Semwal RB, Combrinck S (2016). Myricetin: a dietary molecule with diverse biological activities. Nutrients.

[16] Agraharam G, Girigoswami A, Girigoswami K (2022). Myricetin: a multifunctional flavonol in biomedicine. Curr Pharmacol Rep.

[17] Rahmani AH, Almatroudi A, Allemailem KS (2023). Myricetin: a significant emphasis on its anticancer potential via the modulation of inflammation and signal transduction pathways. Int J Mol Sci.

[18] Benet LZ, Hosey CM, Ursu O (2016). BDDCS, the rule of 5 and drugability. Adv Drug Deliv Rev.

[19] Daneman R, Prat A (2015). The blood-brain barrier. Cold Spring Harb Perspect Biol.

[20] Bogush M, Heldt NA, Persidsky Y (2017). Blood brain barrier injury in diabetes: unrecognized effects on brain and cognition. J Neuroimmune Pharmacol.

[21] Rom S, Zuluaga-Ramirez V, Gajghate S (2019). Hyperglycemia-driven neuroinflammation compromises BBB leading to memory loss in both diabetes mellitus (DM) type 1 and type 2 mouse models. Mol Neurobiol.

[22] Richner M, Ferreira N, Dudele A (2018). Functional and structural changes of the blood-nerve-barrier in diabetic neuropathy. Front Neurosci.

